# Single-Nucleotide Polymorphisms in LEP and LEPR Associated With Breast Cancer Risk: Results From a Multicenter Case–Control Study in Chinese Females

**DOI:** 10.3389/fonc.2022.809570

**Published:** 2022-02-10

**Authors:** Liang Li, Xingchen Meng, Liyuan Liu, Yujuan Xiang, Fei Wang, Lixiang Yu, Fei Zhou, Chao Zheng, Wenzhong Zhou, Shude Cui, Fuguo Tian, Zhimin Fan, Cuizhi Geng, Xuchen Cao, Zhenlin Yang, Xiang Wang, Hong Liang, Shu Wang, Hongchuan Jiang, Xuening Duan, Haibo Wang, Guolou Li, Qitang Wang, Jianguo Zhang, Feng Jin, Jinhai Tang, Liang Li, Shiguang Zhu, Wenshu Zuo, Chunmiao Ye, Gengshen Yin, Zhongbing Ma, Shuya Huang, Zhigang Yu

**Affiliations:** ^1^ Department of Breast Surgery, The Second Hospital, Cheeloo College of Medicine, Shandong University, Jinan, China; ^2^ School of Medicine, Cheeloo College of Medicine, Shandong University, Jinan, China; ^3^ Institute of Translational Medicine of Breast Disease Prevention and Treatment, Shandong University, Jinan, China; ^4^ Shandong Provincial Engineering Laboratory of Translational Research on Prevention and Treatment of Breast Disease, Jinan, China; ^5^ Department of Breast Surgery, Affiliated Tumor Hospital of Zhengzhou University, Zhengzhou, China; ^6^ Department of Breast Surgery, Shanxi Cancer Hospital, Taiyuan, China; ^7^ Department of Breast Surgery, The First Hospital of Jilin University, Changchun, China; ^8^ Department of Breast Center, The Fourth Hospital of Hebei Medical University, Shijiazhuang, China; ^9^ Department of Breast Surgery, Tianjin Medical University Cancer Institute and Hospital, Tianjin, China; ^10^ Department of Thyroid and Breast Surgery, The First Affiliated Hospital of Binzhou Medical University, Binzhou, China; ^11^ Department of Breast Surgery, Cancer Hospital, Chinese Academy of Medical Sciences, Beijing, China; ^12^ Department of General Surgery, Linyi People’s Hospital, Linyi, China; ^13^ Department of Breast Disease Center, Peking University People’s Hospital, Beijing, China; ^14^ Department of General Surgery, Beijing Chaoyang Hospital, Beijing, China; ^15^ Department of Breast Disease Center, Peking University First Hospital, Beijing, China; ^16^ Department of Breast Center, Qingdao University Affiliated Hospital, Qingdao, China; ^17^ Department of Breast and Thyroid Surgery, Weifang Traditional Chinese Hospital, Weifang, China; ^18^ Department of Breast Surgery, The Second Affiliated Hospital of Qingdao Medical College, Qingdao Central Hospital, Qingdao, China; ^19^ Department of General Surgery, The Second Affiliated Hospital of Harbin Medical University, Harbin, China; ^20^ Department of Breast Surgery, The First Affiliated Hospital of China Medical University, Shenyang, China; ^21^ Department of General Surgery, Nanjing Medical University Affiliated Cancer Hospital, Cancer Institute of Jiangsu Province, Nanjing, China; ^22^ Department of Breast and Thyroid Surgery, Zibo Central Hospital, Zibo, China; ^23^ Department of Breast Surgery, Yantai Yuhuangding Hospital, Yantai, China; ^24^ Department of Breast Cancer Center, Shandong Cancer Hospital, Jinan, China

**Keywords:** breast cancer risk, leptin, LEPR, single-nucleotide polymorphisms, case–control study

## Abstract

**Background:**

Leptin (LEP) plays a physiological role through its specific receptor (LEPR) and is involved in the occurrence and development of breast cancer. Our current study aimed at determining the influence of single-nucleotide polymorphisms (SNPs) in the genes coding for LEP and LEPR on breast cancer risk.

**Methods:**

In the present study, 963 breast cancer cases and 953 controls were enrolled. Five SNPs of LEP and two of LEPR were chosen to evaluate the correlation of selected SNPs with breast cancer susceptibility among women in northern and eastern China. Analyses were further stratified by body mass index (BMI), waist–hip rate (WHR), estrogen receptor, and progesterone receptor status. The expression patterns of risk variant-associated genes were detected by expression quantitative trait locus (eQTL) analysis with eQTLGen and The Cancer Genome Atlas database.

**Results:**

There were significant differences between breast cancer cases and control groups in the menopausal status and family history of breast cancer. Two SNPs (rs1137101 and rs4655555) of the LEPR gene decreased overall breast cancer risk, and other five SNPs showed no significant association with breast cancer risk. rs1137101 (GA vs. GG; adjusted OR = 0.719, 95% CI = 0.578–0.894, p = 0.003) and rs4655555 (TT vs. AA; adjusted OR = 0.574, 95% CI = 0.377–0.873, p = 0.009) significantly decreased breast cancer risk after Bonferroni correction for multiple testing. In subgroup analyses, the GA and GA + AA genotypes of LEPR rs1137101 associated with decreased breast cancer risk in the subgroup of BMI ≤ 24 kg/m^2^ or WHR ≥ 0.85 after Bonferroni correction. Furthermore, we found that the expressions of rs4655555-associated gene LEPR and leptin receptor overlapping transcript (LEPROT) were upregulated in breast cancer tumor tissues compared with adjacent normal tissues, and a higher expression of LEPR in tumor tissues was correlated with poor prognosis of breast cancer patients using The Cancer Genome Atlas Breast Invasive Carcinoma (TCGA-BRCA) data.

**Conclusion:**

Our study demonstrated that the polymorphisms rs1137101 and rs4655555 located in the LEPR gene decreased breast cancer risk in Chinese females, which might be a research-worthy bio-diagnostic marker and applied for early prediction and risk assessment of breast cancer.

## Introduction

According to Global Cancer Statistics 2021, breast cancer is the most commonly diagnosed cancer worldwide, which accounts for 30% of all new cancers in women. Furthermore, breast cancer is the second leading cause of cancer death among women (1). China is undergoing cancer transition with an increasing burden of breast cancer, and female breast cancer patients took up 18.41% of breast cancer deaths across the world (2). Breast cancer incidence rates continue to increase by about 0.5% per year, which is attributed at least in part to continued declines in fertility rate and increased body weight (3). There are varieties of factors that can increase the risk of breast cancer, including family history of breast cancer, breastfeeding, secondhand smoke, eating habits, obesity, and diabetes mellitus (4). Based on larger observational studies, obesity is associated with a higher risk of developing breast cancer, particularly in postmenopausal women (5, 6). Obesity induces the dysfunction of adipocyte and changes the expression levels of adipokines and hormones, which will promote the progress of obesity related-tumors (7). A high expression of leptin (LEP) and low levels of adiponectin in obese patients were identified associated with the occurrence of breast cancer (8).

Leptin (LEP), the circulating product of the obesity gene, is a 16-kDa glycoprotein expressed and secreted primarily by the adipocyte. LEP plays an important role in body weight homeostasis by influencing food intake and energy expenditure and maintaining constant energy stores (9). In addition to the regulation of body weight, LEP was also identified to be involved in insulin resistance, cancer cell inflammation, oxidative stress, cell proliferation, apoptosis, angiogenesis, and antitumor immune regulation (10, 11). Leptin exerts its biological action majorly through binding to and activating the leptin receptors (LEPR) (12) and the gene encoding LEPR overlapping transcript (LEPROT).

The expressions of LEP and LEPR were associated with enhanced cell proliferation and angiogenesis in both benign and malignant breast epithelial cells (13–17). Higher circulating leptin concentrations were significantly associated with an increased risk of breast cancer (18). Previous studies indicated that LEPROT could negatively regulate the cell surface expression of LEPR and the silencing LEPROT expression in the mouse hypothalamic arcuate nucleus prevented the development of high-fat-diet-induced obesity (19). In contrast, LEPROT was found to activate the JAK/STAT pathway and may facilitate cancer development (20). A recent study identified an aberrant expression of LEPROT in 78.9% cancers compared with corresponding normal tissues (21).

Studies have identified genetic variants of LEP and LEPR correlated with susceptibility of various malignant tumors, including breast cancer (22, 23). Several single-nucleotide polymorphisms (SNPs) of LEP and LEPR were found correlated with breast cancer risk, including LEP-2548G/A (rs7799039), LEPR K109R (rs1137100), and LEPR Q223R (rs1137101) (24–27), but the results are not exactly consistent. Based on these results, in the current study, we selected 7 polymorphisms located in the LEP and LEPR genes and identified the association with breast cancer risk in northern and eastern Chinese Han females by conducting a multicenter case–control study. This exploration could further provide a research basis for discovering pathogenic targets of breast cancer prevention.

## Materials and Methods

### Participants

Characteristics of the study participants have been reported previously (4). Briefly, participants in the case group were Han ethnic female patients aged 25 to 70 years, who had newly diagnosed, histologically confirmed breast cancer and were recruited at 21 hospitals located in 11 provinces of northern and eastern China between April 2012 and April 2013. The control group comprised age-matched (± 3 years) volunteers recruited at the same hospital who were examined within 2 months of the case group and were confirmed as being breast cancer free by negative physical and imaging findings. Participants with other malignant tumors were excluded from the study. The ethics committee of the Second Hospital, Cheeloo College of Medicine, Shandong University, had approved this study, and all participants signed informed consent.

Personal information and samples from each participant were obtained after signing the informed consent. The demographic information and lifestyle habits of the participants, clinical data including age, body mass index (BMI), waist–hip rate (WHR), personal medical history, family backgrounds, the clinical examination results of visual examination, palpation, and related diagnostic tests such as breast ultrasound and mammography were documented. The statuses of the estrogen receptor (ER) and progesterone receptor (PR) were determined by immunohistochemical staining and obtained from the patients’ medical records. According to the American Society of Clinical Oncology/College of American Pathologists (2020) guideline recommendations, samples with 1% to 100% of tumor nuclei positive for ER or PR are interpreted as positive (28). For each participant, a 4-ml non-fasting blood sample was collected using an EDTA vacutainer (Becton Dickinson, New York). Each blood sample was stored vertically in a freezer at -80°C after sedimentation.

### Genotyping and Laboratory Methods

Blood DNA was extracted using the Wizard Genomic DNA Purification Kit (Promega, Madison, USA). According to previous studies, 7 SNPs were reported in relation with obesity, weight, or cancer risk including rs10244329 (29), rs10954173 (30), rs2167270 (31), rs3828942 (29), and rs4731426 (32) of LEP and rs1137101 (33) and rs4655555 (34) of LEPR (**Supplementary Table 1**) with a minor allelic frequency (MAF) > 5% according to the dbSNP database (https://www.ncbi.nlm.nih.gov/snp/), which were selected for further analysis. All participants were genotyped using the Sequenom MassARRAY SNP system (CapitalBio Technology, Beijing, China), as previously described (35).

### Statistical Analysis

SPSS 26.0 statistical software (IBM, New York) was used to analyze the data. Among them, χ^2^ tests were used to compare the differences in demographic and lifestyle data between the case and control groups. A population representative was detected using Hardy–Weinberg equilibrium (HWE) in the control group. Unconditional logistics regression was used to assess the co-dominant (heterozygous or mutant homozygous vs. wild-type homozygous), dominant (heterozygous and mutant homozygous vs. wild-type homozygous), and recessive (mutant homozygous vs. wild-type homozygous and heterozygous) models of genetic variants and breast cancer risk. Odds ratios (OR) with 95% confidence interval (95% CI) were estimated after adjustment for menstrual status and family history of breast cancer. Subjects were further stratified into subgroups according to BMI, WHR, ER, and PR statuses. Bonferroni correction was used to adjust for multiple testing, and the level of significance was set at α < 0.01 (0.05/5) for testing the five loci of LEP and α < 0.025 (0.05/2) for testing the two loci of LEPR.

The eQTLGen database was used to identify affected genes related to the risk SNPs (36). The expressions of related genes in paired breast tumor tissue and normal tissue (n = 112) of The Cancer Genome Atlas (TCGA) database (https://portal.gdc.cancer.gov/) were further analyzed by using the two-tailed paired Wilcoxon rank-sum test. The association between related genes and the survival rate of breast cancer patients were analyzed by the Kaplan–Meier survival analysis based on TCGA Breast Invasive Carcinoma (TCGA-BRCA) data. The curves were generated with an optimum cutoff value for LEPR or LEPROT expression. A p-value < 0.05 was considered as statistically significant unless otherwise specified.

## Results

### General Demographic Characteristics of Participants

In this study, we enrolled 963 controls and 953 cases. The general demographic characteristics of the participants are presented in **Table 1**. The menstrual status and family history of breast cancer showed significant differences between the case and control groups (p < 0.05). There was no significant difference for other factors between the two groups.

**Table 1:** Demographic characteristics of participants.

Variables*^a^*	Control, n (%)	Case, n (%)	χ^2^	p-value*^b^*
**Age, y**			3.563	0.468
25–34	76 (7.9)	62 (6.5)		
35–44	329 (34.2)	30 (31.7)		
45–54	352 (36.6)	364 (38.2)		
55–64	183 (19.0)	20 (21.0)		
65–70	23 (2.4)	25 (2.6)		
**Age at menarche, y**			0.924	0.630
7–11	16 (1.7)	11 (1.2)		
12–13	231 (24.4)	223 (24.0)		
≥14	699 (73.9)	695 (74.8)		
**Menstrual status**			6.251	**0.012**
Premenstrual	665 (71.9)	614 (66.5)		
Postmenstrual	260 (28.1)	309 (33.5)		
**Family history of breast cancer**			9.556	**0.020**
No	921 (96.2)	884 (93.1)		
Yes	36 (3.8)	66 (6.9)		
**BMI, kg/m^2^**			1.725	0.189
≤24	469 (50.2)	432 (47.2)		
>24	465 (49.8)	484 (52.8)		
**WHR**			2.534	0.111
<0.85	485 (56.5)	420 (52.6)		
≥0.85	374 (43.5)	379 (47.4)		

BMI, body mass index; WHR, waist–hip rate.

a The data were presented in the form of classified variables.

b p-value was calculated by the χ^2^ test, and p < 0.05 was statistically significant (Bold value).

### Genotype Distribution of LEP/LEPR and Breast Cancer Risk

The association of LEP and LEPR SNP genotypes with breast cancer risk after adjustments for risk factors, including menstrual status and family history of breast cancer, is shown in **Table 2**. All SNPs were consistent with HWE in the control group (p > 0.05). Among the 7 SNPs, LEPR rs1137101 showed a significantly decreased breast cancer risk under the dominant genetic model (GA + AA vs. GG, adjusted OR = 0.722, 95% CI = 0.584–0.893, p = 0.003) and co-dominant genetic model (GA vs. GG, adjusted OR = 0.719, 95% CI = 0.578–0.894, p = 0.003). For LEPR rs4655555, a significant association with decreased breast cancer risk was also identified in the co-dominant genetic model (TT vs. AA, adjusted OR = 0.574, 95% CI = 0.377–0.873, p = 0.009) and recessive model (TT vs. TA + AA, adjusted OR = 0.595, 95% CI = 0.394–0.899, p = 0.014). However, the other five SNPs showed no significant association with overall breast cancer risk.

**Table 2:** Genotype distribution of LEP/LEPR and breast cancer risk.

Genotypes	Control, n (%)	Case, n (%)	OR (95% CI)*^a^*	p-value	HWE*^b^*
**LEP rs10244329**					0.996
**Co-dominant**					
AA	533 (56.5)	526 (56.2)	1 (ref.)		
TA	352 (37.3)	368 (39.3)	1.027 (0.846–1.246)	0.791	
TT	59 (6.3)	42 (4.5)	0.774 (0.506–1.184)	0.238	
**Dominant**					
AA	533 (56.5)	526 (56.2)	1 (ref.)		
TA+TT	411 (43.5)	410 (43.8)	0.992 (0.823–1.196)	0.936	
**Recessive**					
TA+AA	885 (93.8)	894 (94.6)	1 (ref.)		
TT	59 (6.3)	42 (4.5)	0.766 (0.504–1.163)	0.211	
**LEP rs10954173**					0.999
**Co-dominant**					
GG	609 (63.4)	599 (62.9)	1 (ref.)		
GA	312 (32.5)	324 (34.0)	1.046 (0.859–1.274)	0.652	
AA	40 (4.2)	30 (3.1)	0.804 (0.489–1.323)	0.391	
**Dominant**					
GG	609 (63.4)	599 (62.9)	1 (ref.)		
GA+AA	352 (36.6)	354 (37.1)	1.020 (0.843–1.233)	0.842	
**Recessive**					
GA+GG	921 (95.8)	923 (96.8)	1 (ref.)		
AA	40 (4.2)	30 (3.2)	0.792 (0.484–1.296)	0.353	
**LEP rs2167270**					0.839
**Co-dominant**					
GG	615 (63.9)	608 (63.8)	1 (ref.)		
GA	312 (32.4)	315 (33.1)	1.016 (0.834–1.238)	0.875	
AA	35 (3.6)	30 (3.2)	0.945 (0.566–1.579)	0.830	
**Dominant**					
GG	615 (63.9)	608 (63.8)	1 (ref.)		
GA+AA	347 (36.1)	345 (36.2)	1.090 (0.834–1.222)	0.926	
**Recessive**					
GA+GG	927 (96.4)	923 (96.9)	1 (ref.)		
AA	35 (3.6)	30 (3.1)	0.94 (0.565–1.564)	0.812	
**LEP rs3828942**					0.977
**Co-dominant**					
AA	548 (57.1)	538 (56.5)	1 (ref.)		
AG	353 (36.8)	372 (39.0)	1.044 (0.861–1.266)	0.659	
GG	59 (6.1)	43 (4.5)	0.798 (0.523–1.217)	0.295	
**Dominant**					
AA	548 (57.1)	538 (56.5)	1 (ref.)		
AG+GG	412 (42.9)	415 (43.5)	1.011 (0.840–1.217)	0.907	
**Recessive**					
AG+AA	901 (93.9)	910 (95.5)	1 (ref.)		
GG	59 (6.1)	43 (4.5)	0.784 (0.518–1.188)	0.251	
**LEP rs4731426**					0.999
**Co-dominant**					
CC	559 (58.2)	549 (57.6)	1 (ref.)		
GC	347 (36.2)	365 (38.3)	1.046 (0.863–1.268)	0.648	
GG	54 (5.6)	39 (4.1)	0.812 (0.522–1.262)	0.354	
**Dominant**					
CC	559 (58.2)	549 (57.6)	1 (ref.)		
GC+GG	401 (41.8)	404 (42.4)	1.017 (0.844–1.224)	0.862	
**Recessive**					
GC+CC	906 (94.4)	914 (95.9)	1 (ref.)		
GG	54 (5.6)	39 (4.1)	0.797 (0.516–1.231)	0.307	
**LEPR rs1137101**					0.663
**Co-dominant**					
GG	691 (71.9)	741 (77.8)	1 (ref.)		
GA	252 (26.2)	197 (20.7)	0.719 (0.578–0.894)	**0.003**	
AA	18 (1.9)	14 (1.5)	0.768 (0.375–1.574)	0.472	
**Dominant**					
GG	691 (71.9)	741 (77.8)	1 (ref.)		
GA+AA	270 (28.1)	211 (22.2)	0.722 (0.584–0.893)	**0.003**	
**Recessive**					
GA+GG	943 (98.1)	938 (98.5)	1 (ref.)		
AA	18 (1.9)	14 (1.5)	0.832 (0.407–1.701)	0.614	
**LEPR rs4655555**					0.650
**Co-dominant**					
AA	548 (57.0)	577 (60.7)	1 (ref.)		
TA	349 (36.3)	334 (35.2)	0.909 (0.748–1.104)	0.335	
TT	65 (6.8)	39 (4.1)	0.574 (0.377–0.873)	**0.009**	
**Dominant**					
AA	548 (57.0)	577 (60.7)	1 (ref.)		
TA+TT	414 (43.0)	373 (39.3)	0.855 (0.710–1.031)	0.101	
**Recessive**					
TA+AA	897 (93.2)	911 (95.9)	1 (ref.)		
TT	65 (6.8)	39 (4.1)	0.595 (0.394–0.899)	**0.014**	

CI, confidence interval; HWE, Hardy–Weinberg equilibrium; OR, odds ratio; ref., reference.

a Adjusted for menstrual status and family history of breast cancer.

b p-value for Hardy–Weinberg equilibrium.

Bonferroni correction, significant if α < 0.025 (in bold).

### Associations Between LEP/LEPR Polymorphisms and Risk of ER+/PR+ or ER-/PR- Breast Cancer Patients

Among the 953 cases, 848 (89.0%) patients had explicit joint ER and PR statuses. Overall, 572 (60.0%) cases were ER+/PR+, 189 (19.8%) cases were ER-/PR-, 72 (7.6%) cases were ER+/PR−, and 15 (1.6%) cases were ER-/PR+. Due to the limited sample size, we excluded ER+/PR- and ER-/PR+ cases for further analysis. The association between the genotypes of LEP/LEPR and the risk of ER+/PR+ or ER-/PR- cases is shown in **Table 3** and **Supplementary Table 2**. LEPR rs1137101 decreased ER+/PR+ breast cancer risk in the dominant genetic model (GA + AA vs. GG, adjusted OR = 0.727, 95% CI = 0.568–0.930, p = 0.011) and the co-dominant genetic model (GA vs. GG, adjusted OR = 0.739, 95% CI = 0.575–0.951, p = 0.019). For LEPR rs4655555, a significant association with decreased ER+/PR+ breast cancer risk in the co-dominant genetic model (TT vs. AA, adjusted OR = 0.533, 95% CI = 0.321–0.884, p = 0.015) and the recessive model (TT vs. TA + AA, adjusted OR = 0.562, 95% CI = 0.341–0.925, P = 0.023) was identified.

**Table 3:** The association of LEP/LEPR genetic variations with risk of ER+/PR+ and ER-/PR- breast cancer.

Genotypes	ER+/PR+				ER-/PR-			
	Control	Case	OR	p- value	Control	Case	OR	p- value
	n (%)	n (%)	(95% CI)*^a^*		n (%)	n (%)	(95% CI)*^a^*	
**rs1137101**								
**Co-dominant**								
GG	691 (71.9)	444 (77.8)	1 (ref.)		691 (71.9)	149 (78.8)	1 (ref.)	
GA	252 (26.2)	121 (21.2)	0.739	**0.019**	252 (26.2)	37 (19.6)	0.669	0.049
			(0.575–0.951)				(0.449–0.998)	
AA	18 (1.9)	6 (1.1)	0.544	0.204	18 (1.9)	3 (1.6)	0.849	0.796
			(0.212–1.393)				(0.245–2.940)	
**Dominant**								
GG	691 (71.9)	444 (77.8)	1 (ref.)		691 (71.9)	149 (78.8)	1 (ref.)	
GA+AA	270 (28.1)	127 (22.2)	0.727	**0.011**	270 (28.1)	40 (21.2)	0.681	0.052
			(0.568–0.930)				(0.462–1.003)	
**Recessive**								
GA+GG	943 (98.1)	565 (98.9)	1 (ref.)		943 (98.1)	186 (98.4)	1 (ref.)	
AA	18 (1.9)	6 (1.1)	0.585	0.263	18 (1.9)	3 (1.6)	0.933	0.912
			(0.229–1.496)				(0.270–3.222)	
**rs4655555**								
**Co-dominant**								
AA	548 (57.0)	352 (61.9)	1 (ref.)		548 (57.0)	112 (59.3)	1 (ref.)	
TA	349 (36.3)	195 (34.3)	0.869	0.221	349	69	0.967	0.848
			(0.693–1.088)		(36.3)	(36.5)	(0.689–1.358)	
TT	65 (6.8)	22 (3.9)	0.533	**0.015**	65 (6.8)	8 (4.2)	0.613	0.211
			(0.321–0.884)				(0.284–1.320)	
**Dominant**								
AA	548 (57.0)	352 (61.9)	1 (ref.)		548 (57.0)	112 (59.3)	1 (ref.)	
TA+TT	414 (43.0)	217 (38.1)	0.815	0.065	414 (43.0)	77 (40.7)	0.910	0.573
			(0.657–1.012)				(0.656–1.262)	
**Recessive**								
TA+AA	897 (93.2)	547 (96.1)	1 (ref.)		897 (93.2)	181 (95.8)	1 (ref.)	
TT	65 (6.8)	22 (3.9)	0.562	**0.023**	65 (6.8)	8 (4.2)	0.620	0.216
			(0.341–0.925)				(0.291–1.322)	

CI, confidence interval; OR, odds ratio; ref., reference.

a Adjusted for menstrual status and family history of breast cancer.

Bonferroni correction, significant if α < 0.025 (in bold).

### The Association Between LEP/LEPR Genotypes and Breast Cancer Risk According to BMI and/or WHR

We further performed stratified analysis to determine the association between LEP/LEPR polymorphisms and breast cancer risk according to obesity indicators including BMI and WHR (**Table 4** and **Supplementary Tables 3, 4**).

**Table 4:** The association of LEP/LEPR genetic variations with breast cancer risk according to BMI or WHR category.

Genotypes	BMI								WHR							
	≤24 kg/m^2^				>24 kg/m^2^				<0.85				≥0.85			
	Control	Case	OR	p-value	Control	Case	OR	p-value	Control	Case	OR	p-value	Control	Case	OR	p-valu
	n (%)	n (%)	(95%CI)*^a^*		n (%)	n (%)	(95% CI)*^a^*		n (%)	n (%)	(95% CI)*^a^*		n (%)	n (%)	(95%CI)*^a^*	e
**rs1137101**																
**Co-dominant**																
GG	327	335	1 (ref.)		342	381	1 (ref.)		348	322	1 (ref.)		261	307	1 (ref.)	
	(69.9)	(77.5)			(73.7)	(78.9)			(71.8)	(76.8)			(70.2)	(81.0)		
GA	130	90	0.657	**0.009**	115	96	0.750	0.073	124	92	0.782	0.125	107	67	0.515	**0.000**
	(27.8)	(20.8)	(0.478–0.902)		(24.8)	(19.9)	(0.548–1.027)		(25.6)	(22.0)	(0.571–1.071)		(28.8)	(17.7)	(0.361–0.736)	
AA	11	7	0.743	0.554	7	6	0.740	0.592	13	5	0.460	0.150	4	5	0.961	0.953
	(2.4)	(1.6)	(0.278–1.986)		(1.5)	(1.2)	(0.245–2.231)		(2.7)	(1.2)	(0.160–1.323)		(1.1)	(1.3)	(0.253–3.650)	
**Dominant**																
GG	327	335	1 (ref.)		342	381	1 (ref.)		348	322	1 (ref.)		261	307	1 (ref.)	
	(69.9)	(77.5)			(73.7)	(78.9)			(71.8)	(76.8)			(70.2)	(81.0)		
GA+AA	141	97	0.663	**0.009**	122	102	0.750	0.066	137	97	0.754	0.070	111	72	0.533	**0.000**
	(30.1)	(22.5)	(0.487–0.902)		(26.3)	(21.1)	(0.552–1.019)		(28.2)	(23.2)	(0.555–1.023)		(29.8)	(19.0)	(0.376–0.755)	
**Recessive**																
GA+GG	457	425	1 (ref.)		457	477	1 (ref.)		472	414	1 (ref.)		368	374	1 (ref.)	
	(97.6)	(98.4)			(98.5)	(98.8)			(97.3)	(98.8)			(98.9)	(98.7)		
AA	11	7	0.825	0.701	7	6	0.789	0.673	13	5	0.489	0.182	4	5	1.119	0.869
	(2.4)	(1.6)	(0.310–2.198)		(1.5)	(1.2)	(0.262–2.374)		(2.7)	(1.2)	(0.171–1.400)		(1.1)	(1.3)	(0.295–4.240)	
**rs4655555**																
**Co-dominant**																
AA	259	252	1 (ref.)		269	305	1 (ref.)		281	253	1 (ref.)		198	235	1 (ref.)	
	(55.2)	(58.3)			(58.0)	(63.3)			(58.1)	(60.4)			(52.9)	(62.3)		
TA	164	157	0.929	0.615	176	162	0.853	0.258	168	151	0.989	0.937	152	128	0.721	0.037
	(35.0)	(36.4)	(0.696–1.239)		(37.9)	(33.6)	(0.648–1.123)		(34.7)	(36.0)	(0.744–1.314)		(40.6)	(34.0)	(0.530–0.981)	
TT	46	22	0.460	**0.006**	19	15	0.736	0.394	35	15	0.453	**0.015**	24	14	0.517	0.062
	(9.8)	(5.1)	(0.264–0.800)		(4.1)	(3.1)	(0.363–1.490)		(7.2)	(3.6)	(0.239–0.860)		(6.4)	(3.7)	(0.258–1.034)	
**Dominant**																
AA	259	252	1 (ref.)		269	305	1 (ref.)		281	253	1 (ref.)		198	235	1 (ref.)	
	(55.2)	(58.3)			(58.0)	(63.3)			(58.1)	(60.4)			(52.9)	(62.3)		
TA+TT	210	179	0.826	0.171	195	177	0.841	0.205	203	166	0.894	0.422	176	142	0.693	**0.016**
	(44.8)	(41.5)	(0.629–1.086)		(42.0)	(36.7)	(0.644–1.099)		(41.9)	(39.6)	(0.681–1.175)		(47.1)	(37.7)	(0.515–0.933)	
**Recessive**																
TA+AA	423	409	1 (ref.)		445	467	1 (ref.)		449	404	1 (ref.)		350	363	1 (ref.)	
	(90.2)	(94.9)			(95.9)	(96.9)			(92.8)	(96.4)			(93.6)	(96.3)		
TT	46	22	0.474	**0.007**	19	15	0.780	0.486	35	15	0.455	**0.015**	24	14	0.587	0.127
	(9.8)	(5.1)	(0.276–0.814)		(4.1)	(3.1)	(0.388–1.568)		(7.2)	(3.6)	(0.242–0.856)		(6.4)	(3.7)	(0.296–1.163)	

CI, confidence interval; OR, odds ratio; ref., reference.

a Adjusted for menstrual status and family history of breast cancer.

Bonferroni correction, significant if α < 0.025 (in bold).

According to the BMI category, rs1137101 and rs4655555 only showed a significant association with decreased breast cancer risk in the subgroup of BMI ≤ 24 kg/m^2^ (**Table 4**, p < 0.01). In the subgroup of WHR ≥ 0.85, rs1137101 and rs4655555 showed a lower breast cancer risk in the dominant genetic model (**Table 4**, rs1137101, GA + AA vs. GG, adjusted OR = 0.533, 95% CI = 0.376–0.755, p = 0.000; rs4655555, TA + TT vs. AA, adjusted OR = 0.693, 95% CI = 0.515–0.933, p = 0.016). A similar association between rs4655555 and breast cancer risk was also identified in women with WHR < 0.85 (**Table 4**, TT vs. AA, adjusted OR = 0.453, 95% CI = 0.239–0.860, p = 0.015; TT vs. TA+ AA, adjusted OR = 0.455, 95% CI = 0.242–0.856, p = 0.015).

We further conducted a stratified analysis by combining BMI and WHR. The results indicated a similar association between the rs1137101 genotype and decreased breast cancer risk in the BMI ≤ 24.0 kg/m^2^ and WHR ≥ 0.85 subgroup (**Supplementary Table 4**, GA+AA vs. GG, adjusted OR = 0.482, 95% CI = 0.288–0.807, p = 0.006) and BMI > 24.0 kg/m^2^ and WHR ≥ 0.85 subgroup (**Supplementary Table 4**, GA+AA vs. GG, adjusted OR = 0.563, 95% CI = 0.344–0.921, p = 0.022). The TT genotype of rs4655555 showed a decreased breast cancer risk in women of BMI ≤ 24.0 kg/m^2^ and WHR < 0.85 (**Supplementary Table 4**, TT vs. AA, adjusted OR = 0.397, 95% CI = 0.179–0.884, p = 0.024; TT vs. TA+ AA, adjusted OR = 0.389, 95% CI = 0.177–0.852, p = 0.018).

### Expression of rs4655555-Associated Genes in Breast Cancer Tissues

**Figure 1:**
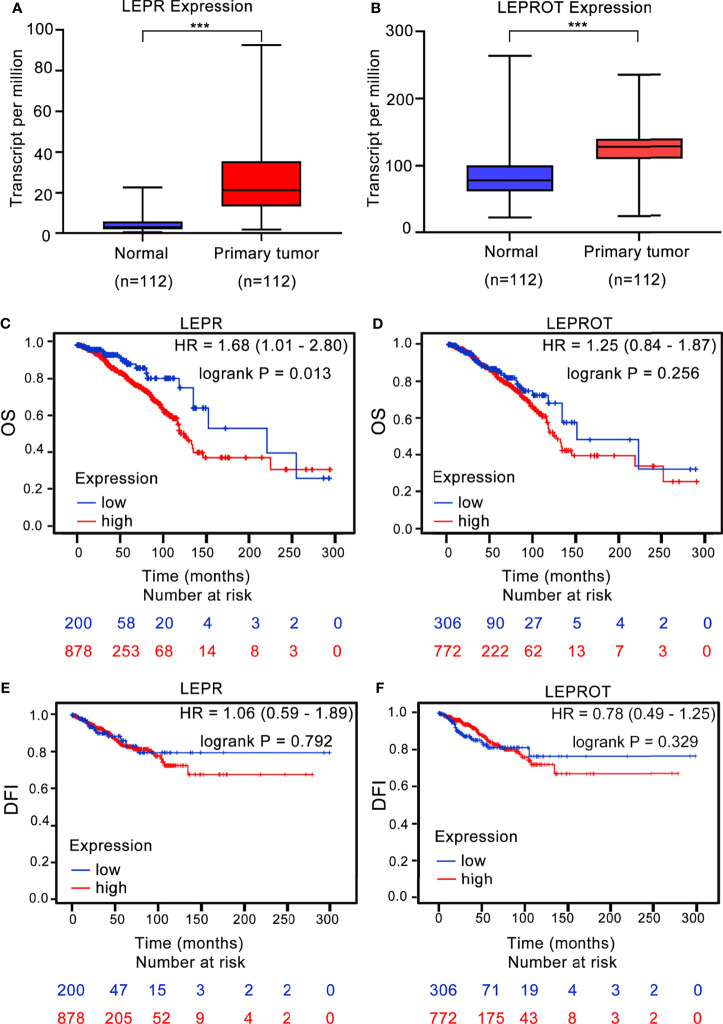
The association between LEPR/LEPROT expression and prognosis of breast cancer patients. The expression of LEPR **(A)** and LEPROT **(B)** in adjacent normal tissues and primary tumor issues of paired TCGA-BRCA data as analyzed by two-tailed paired Wilcoxon’s rank-sum test. ***p-value < 0.001. Kaplan–Meier curves for overall survival (OS) and disease-free interval (DFI) by using TCGA-BRCA data according to high and low LEPR **(C, E)** or LEPROT **(D, F)** gene expression. HR with 95% CI and log-rank p values were calculated. A log-rank p value < 0.05 was considered as statistically significant.

To further identify the downstream associated genes of breast cancer risk-related variants, we found that 2 cis-eQTL genes (LEPR and LEPROT) were associated with rs4655555 based on the eQTLGen database (36) (**Table 5**). The expression pattern of LEPR and LEPROT in breast cancer tissues was analyzed using TCGA data. The results indicated that compared to matched adjacent normal tissues, both LEPR and LEPROT showed a higher expression in breast cancer tumor tissues (p < 0.001, **Figures 1A, B**).

**Table 5:** Identification of rs4655555 associated genes by eQTL analysis.

ID	Chr	Pos (hg19)	ID	Gene symbol	Chr	Pos (hg19)	Z-score	Assessed	Other	Number cohorts	Number samples	FDR	p-value
rs4655555	1	66080269	ENSG00000213625	LEPROT	1	65893980	21.0892	A	T	35	26494	0	9.99E-99
rs4655555	1	66080269	ENSG00000116678	LEPR	1	65996745	14.3964	A	T	34	28588	0	5.45E-47

The association between LEPR or LEPROT level and outcomes of breast cancer patients was further evaluated by Kaplan–Meier survival analysis based on TCGA-BRCA data. A high expression of LEPR is significantly associated with poor OS of breast cancer patients, with HRs (95% CIs) of 1.68 (95% CI: 1.01–2.80) (**Figure 1C**). However, the association between LEPROT expression and OS of breast cancer patients was not significant (**Figure 1D**, HR = 1.25, 95% CI = 0.84–1.87). Furthermore, no significant association between LEPR or LEPROT expression and disease-free interval (DFI) of breast cancer patients was identified (**Figures 1E, F**).

## Discussion

Obesity is a common public health problem nowadays. A number of studies have shown that obesity is associated with the occurrence of breast cancer (5, 6). The molecular mechanisms of the relationship between obesity and breast cancer involve estrogens, insulin, leptin, adiponectin, and inflammatory cytokines. Specifically, the activation of leptin signaling leads to simultaneous activation of multiple oncogenic pathways, leading to increased breast cancer cell proliferation, epithelial–mesenchymal transformation, migration, and invasion (37, 38). Previous studies have shown that elevated leptin levels are associated with aggressiveness and poor prognosis of breast cancer patients (18, 39). In addition, studies also showed that variants of LEP and LEPR gene are associated with breast cancer susceptibility (24–26). In the current study, we recruited 1,616 participants including 963 breast cancer cases and 953 cancer-free controls to assess the correlation between LEP/LEPR polymorphisms and susceptibility of breast cancer. Our current study found a significant association between the LEPR rs1137101 and rs4655555 variants and decreased risk of breast cancer in a Chinese Han population.

The LEPR rs1137101 (Arg223Gln), a missense SNP, has been analyzed for the correlation with cancer risk and development; however, the previous results were not consistent. In the current study, we found that the GA and GA+AA genotypes of rs1137101 were associated with decreased breast cancer risk. Some previous case–control studies reported that the A allele of rs1137101 is a protective factor against cancer occurrence (26, 40, 41), which were consistent with our results, but some studies have shown the opposite results (42, 43). The inconsistent results may be due to differences in race, genetic background, environment, or lifestyle.

We further identified that the genotype of LEPR rs1137101 was associated with decreased breast cancer risk under the dominant genetic model in the subgroup of WHR ≥ 0.85 regardless of BMI by stratified analysis (**Table 4** and **Supplementary Table 4**). The risk of breast cancer in rs1137101 GA carriers was most significantly reduced in women with WHR ≥ 0.85 and normal BMI (**Supplementary Table 4**, BMI ≤ 24.0 kg/m^2^ and WHR ≥ 0.85, adjusted OR = 0.446, 95% CI: 0.264–0.754, p = 0.003), which was the indicator of central obesity. Our previous study indicated that central obesity was positive with ER-/PR- breast cancer risk (44). A similar trend of the GA genotype of rs1137101 was associated with a decreased risk in ER-/PR- breast cancer (**Table 3**), indicating that rs1137101 may participate in mediating the correlation between central obesity and ER-/PR- breast cancer risk. We also found that the GA + AA genotype of rs1137101 was significantly associated with decreased breast cancer risk in women of WHR ≥ 0.85 and BMI > 24.0 kg/m^2^, which may partially explain for the reduced risk of ER+/PR+ cases (**Table 3** and **Supplementary Table 4**). A previous study has shown that serum leptin levels are positively associated with increased expression of ER and PR in breast cancer patients (45). Similarly, LEPR expression was positively correlated with tumor size and ER expression in breast cancer (46). However, the association between rs1137101 genotypes and LEPR expression is still unknown. Therefore, the in-depth mechanism between rs1137101 and breast cancer risk in different hormone receptor statuses needs to be further explored.

To our knowledge, the association between rs465555 and breast cancer susceptibility has not been demonstrated in previous studies. We found that the TT genotype of rs4655555 was associated with decreased overall breast cancer risk. A similar association was identified in the subgroup of BMI ≤ 24.0 kg/m^2^ and BMI ≤ 24.0 kg/m^2^ and WHR < 0.85. Based on the eQTLGen database, we identified 2 cis-eQTL genes of rs4655555, including LEPR and LEPROT, the receptors for leptin action (47). A previous genome-wide association study also identified a strong association between rs4655555 and circulating soluble leptin receptor (sOB-R) levels (34). There is no previous correlation study between rs465555 and LEPROT expression. To evaluate the potential causal function of rs4655555-associated genes in breast cancer risk, based on the TCGA database, we found a higher expression of LEPR and LEPROT in breast cancer tumor tissues compared to that of adjacent normal tissues. Previous studies indicated that leptin signaling played a key role in breast cancer incidence and development, and a higher expression of leptin and LEPR was also validated in breast cancer tissues (48, 49). The expression of LEPR is also necessary for maintaining cancer stem cell-like and metastatic properties in triple-negative breast cancer (50). We further identified that a high expression of LEPR significantly correlated with worse prognosis of breast cancer patients, which suggesting that the rs4655555 variant may affect breast cancer risk and development through regulating the expression of LEPR, and subsequently prognosis. Further confirmatory studies are necessary to validate the regulatory mechanism in variant-associated breast carcinogenesis.

The strengths of this study include the multicenter retrospective study design, the large sample size, the availability of fasting blood samples, and the measurement and examination of relevant indicators in participants using standardized procedures. However, some limitations of this study need to be addressed. First, although the number of participants was relatively large, the sample sizes of some subgroups were small. A larger external multicenter prospective cohort study needs to be further conducted to validate our identification. Furthermore, the correlation between rs1137101, rs4655555, and downstream causal genes needs to be further validated by conducting experimental analyses.

In conclusion, our study provides evidence that rs1137101 and rs4655555 of the LEPR gene are associated with breast cancer susceptibility in Chinese women. In addition, rs1137101 may have the potential to inhibit the occurrence and development of breast cancer in centrally obese women, providing new ideas for the prevention of obesity-associated breast cancer.

## Data Availability Statement

The original contributions presented in the study are included in the article. Further inquiries can be directed to the corresponding authors on reasonable request.

## Ethics Statement

The studies involving human participants were reviewed and approved by the Institutional Review Board of The Second Hospital, Cheeloo College of Medicine, Shandong University. The patients/participants provided their written informed consent to participate in this study.

## Author Contributions

ZGY conceived and designed the study. XM and SH performed statistical analyses. LYL and FW organized the database. LL (1st author), XM, and SH wrote the first draft of the manuscript. LY, FZ, and CZ contributed to the manuscript revision and statistical analyses. YX, WZZ, CY, GY, and ZM contributed to the DNA extraction. SC, FT, ZF, CG, XC, ZLY, XW, HL, SW, HJ, XD, HW, GL, QW, JZ, FJ, JT, LL (27th author), SZ, and WSZ contributed to the collection of the data and biological samples. All authors contributed to the manuscript revision and read and approved the submitted version.

## Funding

This study was funded by the National Natural Science Foundation of China (82072914), Natural Science Foundation of Shandong Province (ZR2019PH016), and National Key Research and Development Program of China (2016YFC0901300).

## Conflict of Interest

The authors declare that the research was conducted in the absence of any commercial or financial relationships that could be construed as a potential conflict of interest.

## Publisher’s Note

All claims expressed in this article are solely those of the authors and do not necessarily represent those of their affiliated organizations, or those of the publisher, the editors and the reviewers. Any product that may be evaluated in this article, or claim that may be made by its manufacturer, is not guaranteed or endorsed by the publisher.
