# Supplementary material for: Single-Nucleotide Polymorphisms in LEP and LEPR Associated With Breast Cancer Risk: Results From a Multicenter Case–Control Study in Chinese Females
Source: Front Oncol. 2022 Feb 10;12:809570. doi: 10.3389/fonc.2022.809570 (PMC8866686; doi:10.3389/fonc.2022.809570)
Supplement: Supplementary file 1 [file Table_1.docx]

Supplementary Material

# Supplementary Tables

**Supplementary Table 1. Description for LEP and LEPR polymorphisms**

| **Gene** | **SNP ID** | **Chr.** | **Chr. Pos** | **Functional Consequence** | **Base change** | **MAF** |
| --- | --- | --- | --- | --- | --- | --- |
| LEP | rs10244329 | 7 | 128248636 | Intron Variant | A>T | 0.466 |
| LEP | rs10954173 | 7 | 128251387 | Intron Variant | G>A | 0.274 |
| LEP | rs2167270 | 7 | 128241296 | 5-Prime UTR Variant | G>A | 0.341 |
| LEP | rs3828942 | 7 | 128254252 | Intron Variant | G>A | 0.424 |
| LEP | rs4731426 | 7 | 128242017 | Intron Variant | G>A, C, T | 0.457 |
| LEPR | rs1137101 | 1 | 65592830 | Missense Variant | A>G, T | 0.416 |
| LEPR | rs4655555 | 1 | 65614586 | Intron Variant | T>A, G | 0.293 |

Abbreviations: MAF, minor allele frequency; SNP, single nucleotide polymorphism.
